# Comparative evaluation of efficacy of subgingivally delivered 1.2% Atorvastatin and 1.2% Simvastatin in the treatment of intrabony defects in chronic periodontitis: a randomized controlled trial

**DOI:** 10.15171/joddd.2017.004

**Published:** 2017-03-15

**Authors:** Santosh S. Martande, Minal Kumari, A. R. Pradeep, Sonender Pal Singh, Deepak Kumar Suke

**Affiliations:** ^1^Department of Periodontics, Dr. D.Y. Patil Dental College and Hospital, Pimpri, Pune, India; ^2^Department of Periodontics, Vydehi Institute of Dental Sciences and Research Centre, Bangalore, India; ^3^Department of Periodontics, Government Dental College & Research Institute, Bangalore, India

**Keywords:** Atorvastatin, bone regeneration, clinical trial, scaling and root planing, Simvastatin

## Abstract

***Background.*** Statins are the recently evolved agents that aid in periodontal regeneration and ultimately in attaining periodontal health. Atorvastatin (ATV) and Simvastatin (SMV) are specific competitive inhibitors of 3-hydroxy-2-methyl-glutaryl coenzyme A reductase. The current study was conducted to compare the effectiveness of 1.2% ATV and 1.2% SMV, in addition to scaling and root planing (SRP), in the treatment of intrabony defects in subjects with chronic periodontitis.

***Methods.*** Ninety-six individuals were categorized into three treatment groups: SRP plus 1.2% ATV, SRP plus 1.2% SMV and SRP plus placebo. Clinical parameters of full-mouth plaque index (PI), modified sulcus bleeding index (mSBI), probing depth (PD), and relative attachment level (RAL) were recorded at baseline before SRP and at 3, 6 and 9 months. Bone fill was assessed using percentage radiographic defect depth reduction at baseline, 6 months and 9 months.

***Results.*** Both ATV and SMV showed significant PD reduction and RAL gain than placebo. ATV group showed greater mean PD reduction and mean RAL gain as compared to SMV group at 3, 6 and 9 months. Furthermore, ATV group sites exhibited a significantly greater percentage of radiographic defect depth reduction (33.23 ± 3.11%; 34.84 ± 3.07%) as compared to SMV (30.39 ± 3.36%; 32.15 ± 3.37%) at 6 and 9 months.

***Conclusion.*** ATV resulted in greater improvements in clinical parameters with higher percentage of radiographic defect depth reduction as compared to SMV in the treatment of intrabony defects in CP subjects.

## Introduction

Chronic periodontitis (CP) is a multi-factorial infectious disease that occurs as a result of the host immune inflammatory response to pathogenic microorganisms, leading to the destruction of periodontal tissues, bone resorption and ultimately tooth loss.^1^The primary goal of periodontal treatment is regeneration of periodontal tissue and maintenance of the architecture and function of the periodontium.^2^ Recently various bone replacement materials along with biologic mediators have been used to enhance the quality and quantity of bone to be regenerated.^3^

Statins, inhibitors of 3-hydroxy-3-glutaryl-coenzyme A reductase, like simvastatin (SMV) and atorvastatin (ATV), are widely used to lower cholesterol in patients with hyperlipidemia and arteriosclerosis.^4,5^Apart from lipid-lowering properties, statins also possess dynamic properties like anti-inflammatory effects as shown by reduction in MMP-9 and TNF-α levels.^6^They arealso thought to increase angiogenesis and bone formation as recognized by expression of bone anabolic factors such as vascular endothelial growth factor and bone morphogenic protein-2,^7,8^ promising increased bone regeneration.

SMV has been found to have an anti-inflammatory effect when locally delivered in various animal studies and to promote bone regeneration.^9,10^Statin users have been found to have improved periodontal condition as compared to non-statin users.^11,12^Recently, our studies showed that local delivery of statins, 1.2% SMV and 1.2% ATV, into periodontal pocket stimulates a significant increase in the PD reduction, relative attachment (RAL) gain, and improved radiographic defect depth reduction as compared to placebo gel as an adjunct to scaling and root planing (SRP) in the treatment of CP and individuals with type II diabetes and CP.^13-16^

Considering the lipid-lowering properties of various statins, ATV has been found to be more effective compared to SMV in patients with hyperlipidemia.^17^Statins also have antioxidant and anti-atherogenic effects beyond their cholesterol-lowing effect, and ATV is thought to have strong antioxidant and anti-inflammatory potential as compared to SMV.^18,19^

Considering the fact that newer options are arising in the treatment of periodontal disease and various regenerative materials are been introduced, it becomes imperative to evaluate and compare the efficacy of available agents in attaining the goal of periodontal regeneration. To the best of our knowledge, no study has compared the use of 1.2% ATV and 1.2% SMV for the treatment of periodontal intrabony defects (IBD’s). Thus, the aim of the present study was to investigate the additional efficacy of 1.2% ATV and 1.2% SMV gel as local drug delivery (LDD) agents as an adjunct to scaling and root planing (SRP) for the treatment of IBDs in individuals with CP.

## Methods

### Study population

In this longitudinal interventional study with 9-month follow-up, a total of 96 individuals (50 males and 46 females, aged 30 to 50 years) who were diagnosed with moderate to severe CP were selected from the outpatient section of the Periodontics Department, Government Dental College and Research Institute, Bangalore, India. The research protocol was initially submitted to the Institutional Ethical Committee and Review Board of the Government Dental College and Research Institute, Bangalore. After ethical approval, all the individuals were verbally informed, and written informed consent was taken for participation in the study. The study was conducted from February 2013 to November 2013. The study protocol was registered under clinicaltrials.gov with identifier number NCT02060032.

### Selection criteria

Moderate-to-severe CP individuals with PD ≥5 mm or clinical attachment level (CAL) ≥4 mm and vertical bone loss ≥3 mm on intraoral periapical radiographs and no history of antibiotic or periodontal therapy in the preceding 6 months were included. Individuals with acceptable plaque control after SRP were continued in the study protocol for local drug delivery while those with unacceptable plaque control were excluded. Individuals with aggressive periodontitis, systemic conditions affecting the periodontal status, individuals on statin therapy or allergic to any statin constituents, nutritional deficient states or immunocompromised conditions, pregnant and lactating females and smokers or tobacco users in any form were excluded from the study.

The randomization process was carried out by the study designer (ARP) using a computer-generated random table and individuals were randomly assigned to either ATV, SMV or placebo group after subject enrolment. A total of 32 individuals each were randomly allotted to one of the three groups. In the ATV group, the sites were treated with SRP followed by 1.2% ATV gel (1.2 mg/0.1 mL) LDD; in the SMV group, the sites were treated with SRP followed by 1.2 % SMV gel (1.2 mg/0.1 mL) LDD, while in the placebo group, the sites were treated with SRP followed by placebo gel placement. Only one site per subject was enrolled for ATV, SMV or placebo groups. Individuals were blinded for allocation to ATV, SMV or placebo groups. SRP was performed at baseline using ultrasonic scalers and Gracey curettes by the operator (SSM). The individuals were restrained from using any antibiotics or anti-inflammatory agents after SRP or during the subsequent study period. Clinical parameters, including modified sulcus bleeding index^20^ (mSBI), full-mouth plaque score^21^(PI), PD and RAL were recorded at baseline (before the SRP) and at 3, 6 and 9 months using a custom-made acrylic stent and a University of North Carolina no. 15 color-coded periodontal probe (Hu-Friedy, Chicago, IL, USA) to standardize the measurements by the examiner (MK).

### Intra-examiner calibration

Before the start of the study, intra-examiner calibration was achieved by examination of 30 sites twice, 24 h apart. Calibration was accepted if measurements at baseline and 24 h were similar to 1 mm at the 95% level.

### Formulation of 1.2 % ATV and 1.2% SMV gels

After intensive in vitro investigations for optimization and stability, ATV and SMV gels were prepared, as described by previous trials on 1.2% ATV and 1.2% SMV local drug delivery.^13,14^ Methylcellulose in situ gel was prepared as described by Thylin et al.^22^ Briefly, accurately weighed methylcelluslose was added to a required amount of biocompatible solvent to prepare methyl cellulose in situ gel. The vial was heated at 50°C to 60°C and shaken well with a mechanical shaker to obtain a clear solution. Two separate solutions were prepared for ATV and SMV gels. Weighed amounts of ATV and SMV were added to the above solutions and dissolved completely to obtain a homogeneous phase of polymer, solvent and drug. Thus, the ATV and SMV in situ gels were prepared with a concentration of ~1.2%.

### Local drug delivery

For standardization, 10 mL of ATV gel (1.2 mg/0.1 mL) and 10 mL of ATV gel (1.2 mg/0.1 mL) were injected into the periodontal pockets of allocated individuals using a syringe with a blunt cannula until the cannula tip touched the base of the pocket. The subjects were strictly instructed to avoid chewing hard or sticky foods, brushing near the treated areas or using any interdental aids for 1 week. The subjects were asked for any adverse effects at the recall visit. Any supragingival deposits seen were removed at each recall visit.

### Radiographic assessment of intrabony defects (IBD)

IBD depth was measured as the vertical distance between the crest of the alveolar bone and the base of the defect. For standardization purpose, individually customized bite blocks and a parallel-angle technique were used to obtain films as reproducible as possible. The radiographic defect depth reduction was assessed by evaluating the defect depth at baseline and 9 months postoperatively. For assessment, radiographs were scanned with a scanner (HP Scanjet 3c/I, Hewlett Packard, Singapore) at 400 dpi by a masked evaluator (DKS) who was blinded to the surgical procedures performed in subjects. The radiographic IBD depth was measured by a computer-aided software program as used previously in previous studies.^13,14^

### Primary and secondary outcome measures

The primary outcome of the study was radiographic defect depth reduction from baseline to 9 months in all the groups. The secondary outcomes included changes in PD, RAL, mSBI and PI from baseline to 9 months.

### Statistical analysis

Power analysis was performed before the start of the study and an ideal sample size was calculated considering differences of at least 1 mm between the groups for RAL changes in sites with initial PD >6 mm and assuming a standard deviation of 1.0 mm. Based on this analysis, 25 individuals per group would be necessary to provide 90% power at α=0.05 between the null hypothesis and the alternative mean. The results were averaged (mean ± standard deviation) for each clinical and radiographic parameter at all time intervals. Mean changes for the period of 9 months (baseline/9 months) were calculated for all the parameters. Unpaired t-test was used to assess the differences for mean changes for all the parameters for each pair of groups. Repeated-measures ANOVA was used to assess the change for the assessed parameters at all time intervals with the within-subject effect. Statistical analyses were carried out using statistical software (SPSS version 16.0, SPSS Inc., Chicago, IL, USA). Statistical significance was set at P ≤ 0.005.

## Results

**Figure 1.**
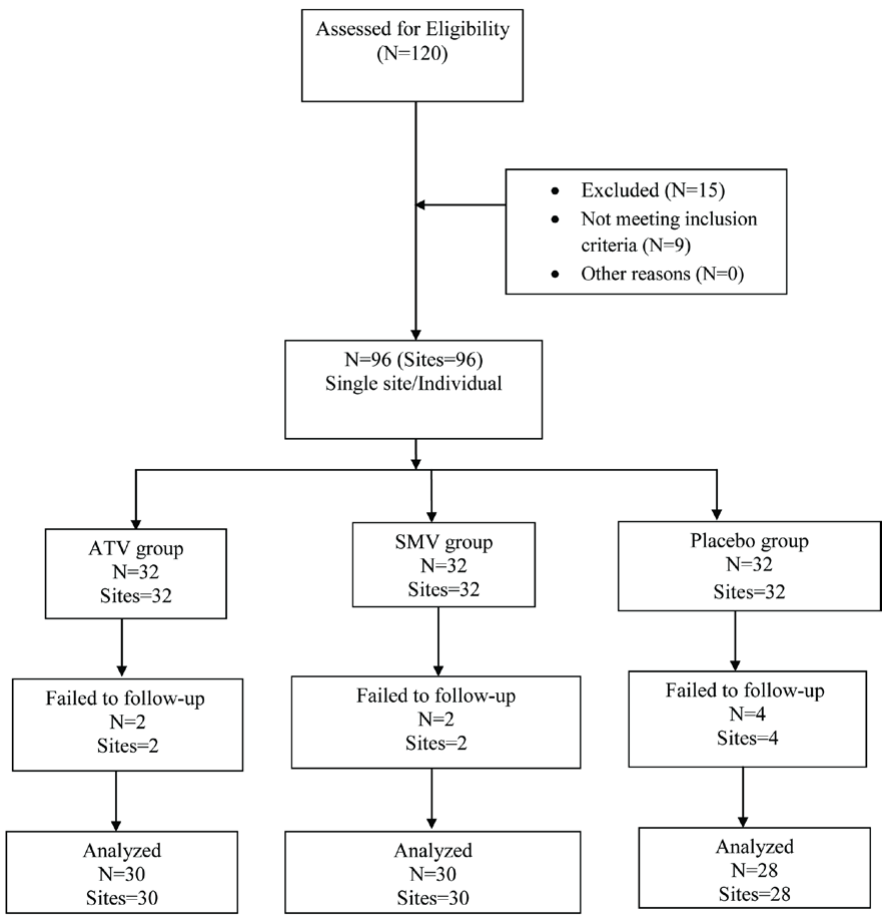
Consort flow chart.

A total of 88 subjects (one site/subject) out of 96 completed the study (Figure 1). Two subjects from the ATV group, 2 from the SMV group and 4 from the placebo group were not followed due to various reasons. All the subjects tolerated the drug well without any complications or adverse reactions. Soft tissues healed within normal limits, and no significant visual differences were noted.

Table 1 demonstrates the full-mouth PI values (mean ± SD), while mean changes in PI for all the groups from baseline to 9 months are presented in Table 3. All the groups showed improvements in full-mouth PI score, but there were no statistically significant differences in full-mouth PI scores between the groups at any visit (Tables 1and3). This indicates that all the groups maintained comparable levels of oral hygiene throughout the study.

**Table 1:** Mean ± SD values of plaque index and mean sulcus bleeding index at all-time intervals for placebo, 1.2% ATV and 1.2% SMV groups

**Parameter**	**Interval**	**1.2% ATV**	**1.2% SMV**	**Placebo**	**P-value**
**PI**	B/L	1.55 ± 0.33	1.59 ± 0.27	1.64 ± 0.29	0.938
	3 months	1.01 ± 0.26	1.03 ± 0.22	1.08 ± 0.30	
	6 months	0.55 ± 0.17	0.57 ± 0.18	0.60 ± 0.21	
	9 months	0.59 ± 0.26	0.60 ± 0.25	0.66 ± 0.22	
**mSBI**	B/L	2.56 ± 0.29	2.52 ± 0.39	2.49 ± 0.24	<0.0001^*^
	3 months	1.21 ± 0.22	1.34 ± 0.31	1.45 ± 0.30	
	6 months	1.05 ± 0.22	1.03 ± 0.28	1.31 ± 0.35	
	9 months	1.02 ± 0.23	1.12 ± 0.32	1.56 ± 0.36	

^*^Statistically significant at P<0.05

**Table 3:** Mean changes in plaque index (PI) and mean sulcus bleeding index (SBI) over a 9-month period in the groups

**Parameter**	**1.2% ATV**	**1.2% SMV**	**Placebo**		**P-value**
**Mean PI change**	0.96 ± 0.40	0.99 ± 0.36	0.98 ± 0.34	Placebo vs 1.2% ATV	0.768
				Placebo vs 1.2% SMV	0.948
				1.2% ATV vs 1.2% SMV	0.936
**Mean mSBI change**	1.54 ± 0.35	1.40 ± 0.43	0.93 ± 0.44	Placebo vs 1.2% ATV	<0.001^*^
				Placebo vs 1.2% SMV	<0.001^*^
				1.2% ATV vs 1.2% SMV	0.647

^*^Statistically significant at P<0.05

Table 1 presents mean ± SD values of mSBI for all the groups at all the intervals while mean changes in mSBI for all the groups from baseline to 9 months are presented in Table 3. mSBI values in all the groups revealed no differences at baseline. But they decreased in the ATV group as compared to the SMV group, which was significant when compared to the placebo group at 6 and 9 months (P <0.05) (Tables 1and 3).

Clinical parameters of PD and RAL also revealed no differences between the groups at baseline. However, ATV group exhibited greater PD reduction and RAL gain as compared to the SMV group, though not statistically significant at 3, 6 and 9 months. Both ATV and SMV groups demonstrated statistically significant PD reductions and RAL gains compared to the placebo group at P < 0.001 (Tables 2and 4).

**Table 2:** Mean ± SD values of probing depth, relative attachment level, intrabony defect depth reduction and percentage of intrabony defect depth reduction at all-time intervals for placebo, 1.2% ATV and 1.2% SMV groups

**Parameter**	**Interval**	**1.2% ATV**	**1.2% SMV**	**Placebo**	**P-value**
**PD**	B/L	7.53 ± 1.35	7.70 ± 1.29	7.63 ± 1.18	
	3 months	5.33 ± 0.92	5.73 ± 0.86	6.46 ± 1.13	
	6 months	4.45 ± 0.77	4.73 ± 0.86	6.08 ± 1.06	
	9 months	4.16 ± 0.86	4.60 ± 0.89	6.38 ± 1.14	<0.0001^*^
**RAL**	B/L	6.70 ± 1.17	6.83 ± 1.14	6.83 ± 1.01	
	3 months	4.46 ± 1.00	4.73 ± 1.01	5.63 ± 0.99	
	6 months	3.36 ± 0.92	3.76 ± 1.30	5.40 ± 1.06	
	9 months	3.23 ± 1.10	3.70 ± 1.20	5.53 ± 1.07	<0.0001^*^
**IBD depth reduction**	B/L	4.86 ± 0.56	4.75 ± 0.50	4.72 ± 0.47	
	6 months	3.24 ± 0.48	3.30 ± 0.44	4.56 ± 0.43	
	9 months	3.17 ± 0.50	3.22 ± 0.43	4.60 ± 0.46	<0.0001^*^
**% IBD depth reduction**	6 months	33.23 ± 3.11	30.39 ± 3.36	3.40 ± 0.43	
	9 months	34.84 ± 3.07	32.15 ± 3.37	2.66 ± 0.39	<0.0001^*^

^*^Statistically significant at P<0.05

**Table 4:** Mean changes in the probing depth, relative attachment level and intrabony defect depth reduction over a 9-month period in the groups

**Parameter**	**1.2% ATV**	**1.2% SMV**	**Placebo**		**P-value**
**Mean PD change**				Placebo vs 1.2% ATV	<0.001^*^
	3.37 ± 1.32	3.10 ± 1.55	1.25 ± 1.24	Placebo vs 1.2% SMV	<0.001^*^
				1.2% ATV vs 1.2% SMV	0.620
**Mean RAL change**				Placebo vs 1.2% ATV	<0.001^*^
	3.46 ± 1.47	3.13 ± 1.56	1.30 ± 1.51	Placebo vs 1.2% SMV	<0.001^*^
				1.2% ATV vs 1.2% SMV	0.674
					
				Placebo vs 1.2% ATV	<0.001^*^
	1.69 ± 0.34	1.53 ± 0.41	0.12 ± 0.10	Placebo vs 1.2% SMV	<0.001^*^
**Mean IBD change**				1.2% ATV vs 1.2% SMV	0.153

^*^Statistically significant at P<0.0

The radiographic parameter IBD showed statistically significant mean reduction of 1.69 ± 0.34 mm at 9 months in the ATV group in comparison to the SMV (1.53 ± 0.41 mm) and placebo groups (0.12 ± 0.10 mm; Table 4).

ATV group sites presented with a significantly greater percentage of radiographic defect depth reduction (33.23 ± 3.11%; 34.84 ± 3.07%) as compared to SMV (30.39 ± 3.36%; 32.15 ± 3.37%) and placebo sites (3.40 ± 0.43%; 2.66 ± 0.39%) at 6 and 9 months, respectively (Table 2and4).

Tables 5and6 show repeated-measures ANOVA results in relation to the assessment of changes in the parameters at different intervals with the within-subject effect. There were statistically significant differences (P < 0.001) in all the parameters (mSBI, PD, RAL and IBD depth) except for PI at different time intervals evaluated.

**Table 5:** Changes in plaque index (PI) and mean sulcus bleeding index (SBI) at different time intervals with the within-subject effect by repeated-measures ANOVA

**Source**	**Type III Sum of Squares**	**df**	**Mean Square**	**F value**	**P value**
**(PI)**					
**Test of Within Individuals Effect**					
**Time**	49.768	3	16.589	309.793	0.0001^*^
**Time x Group**	0.095	6	0.016	0.297	0.938
**Error (Time)**	13.976	261	0.054		
**Test of Between Individuals Effects**					
**Group**	0.262	2	0.131	1.291	0.280
**Error**	8.814	87	0.101		
**(GI)**					
**Test of Within Individuals Effect**					
**Time**	129.405	3	43.135	686.264	0.0001^*^
**Time x Group**	3.610	6	0.602	9.572	0.0001^*^
**Error (Time)**	16.405	261	0.063		
**Test of Between Individuals Effects**					
**Group**	7.443	2	3.722	20.676	0.0001^*^
**Error**	15.660	87	0.180		

^*^Statistically significant at P<0.05

**Table 6:** Changes in probing depth (PD), relative attachment level (RAL), intrabony defect (IBD) depth reduction and % intrabony defect depth reduction at different time intervals with the within-subject effect by repeated-measures ANOVA

**Source**	**Type III Sum of Squares**	**df**	**Mean Square**	**F value**	**P value**
			**(PD)**		
			**Test of Within Individuals Effect**		
**Time**	394.342	3	131.447	303.273	0.0001^*^
**Time x Group**	44.283	6	7.381	17.028	0.0001^*^
**Error (Time)**	113.125	261	0.433		
			**Test of Between Individuals Effects**		
**Group**	109.006	2	54.503	17.664	0.0001^*^
**Error**	268.442	87	3.086		
			**(RAL)**		
			**Test of Within Individuals Effect**		
**Time**	413.211	3	137.737	185.577	0.0001^*^
**Time x Group**	50.072	6	8.345	11.244	0.0001^*^
**Error (Time)**	193.717	261	0.742		
			**Test of Between Individuals Effects**		
**Group**	131.017	2	65.508	25.686	0.0001^*^
**Error**	221.883	87	2.550		
			**(IBD depth)**		
			**Test of Within Individuals Effect**		
**Time**	72.448	2	36.224	997.801	0.0001^*^
**Time x Group**	27.562	4	6.890	189.797	0.0001^*^
**Error (Time)**	6.317	174	0.036		
			**Test of Between Individuals Effects**
**Group**	45.414	2	22.707	36.738	0.0001^*^
**Error**	53.774	87	0.618		

^*^Statistically significant at P<0.05

## Discussion

The present study evaluated and compared the clinical efficacy of 1.2% ATV and 1.2% SMV gels as adjuncts to SRP for the treatment of IBDs and found that 1.2% ATV resulted in greater intrabony defect depth reduction and improvements in clinical parameters as compared to 1.2% SMV.

Statins (ATV and SMV) are competitive inhibitors for HMG-CoA reductase and are mostly used to lower cholesterol, in the treatment of hyperlipidemia.^5^ Systemically delivered ATV has superior kinetics as compared to other statins. In a study to evaluate the pharmacokinetic profile and dose effectiveness of different statins in the reduction of cholesterol, ATV (5 mg) was found to attain target therapeutic concentrations to bring about a 30% reduction in LDL cholesterol (valid surrogate marker) as compared to 10 mg SMV and 40 mg lovastatin.^23^ ATV has been found to be more effective compared to SMV and pravastatin in patients with hyperlipidemia.^17^ ATV at 10, 20, 40 mg doses was found to be more effective compared to other statins in reducing total and LDL cholesterol in comparative dose efficacy study (CURVES study).^24^Statins have antioxidant and antiatherogenic effects beyond their cholesterol-lowering effect and the effects of ATV on reducing oxidative stress were significantly greater compared with those of SMV in individuals with coronary artery disease and type II diabetes.^18,19^A study also showed that compared with SMV, ATV exhibited more anti-inflammatory properties as suggested by markers of oxidative stress and inflammation in patients with type II diabetes.^25^ Thus superior pharmacokinetic properties and potent antioxidant and anti-inflammatory properties can be considered as one of the reasons for superior results in the ATV group as compared to the SMV group.

Locally delivered statins offer obvious advantages of increased concentration, reduced adverse effects and high patient compliance as compared to systemic regimen.^26^ATV and SMVhave been found to have osteoblastic actions in animal and human studies.^13-16^,Fajardo et al^27^in a study found that ATV administration improved periodontal parameters, including PD, CAL and mobility over a 3-month period. Morris et al^28^ showed effective IBD fill and a greater decrease in PD and RAL gain compared to the control group when injectable SMV was used in three-walled periodontal IBDs, class II furcations defects and edentulous alveolar ridges in beagle dogs.

Considering the clinical parameters, a decrease in PD and a gain in RAL are the major clinical outcomes measured to determine the success of any periodontal treatment and there were significant reductions in PD and gain in RAL in both the ATV and SMV groups compared to placebo at all the time intervals. ATV resulted in greater reduction in PD and gain in RAL as compared to SMV, although not statistically significant. Moreover, a decrease in gingival bleeding was also greater in ATV as compared to SMV group, thus suggesting a potent anti-inflammatory action of ATV. A similar anti-inflammatory effect of statins was observed by Lindy et al^11^in patients with CP, who were on systemic statin therapy.

The mean percentage defect depth reduction in the ATV group (34.84 ± 3.07%) was greater than the SMV group (32.15 ± 3.37%) and significantly greater than placebo (2.66 ± 0.39%). The results of this study were comparable to our previous studies in which ATV and SMV were found to enhance clinical and radiographic outcomes in the treatment of intrabony defects in CP and individuals with type II diabetes and CP.^13-16^

Thus the results of this study can be attributed to rather superior pharmacokinetic and functional properties of ATV over SMV, which can pave way for understanding the differences in opinions on the use of either statin in inflammatory periodontal disease. In the current study no adverse effects were observed. However, side effects of the use of statins should always be considered when treating patients with statin local drug delivery.^29^

Further longitudinal, multi-centered studies with larger sample sizes and histological analysis to measure defect bone fill are necessary to validate the result of this trial.

## Conclusion

There were greater improvements in clinical parameters and significant IBD depth reduction with locally delivered ATV as compared with SMV, though not statistically significant, in individuals with CP as an adjunct to SRP. Further clinical, radiographic and histological analyses are needed to confirm the results of the study and evaluate the effect of statins on bone function and remodeling.

## Acknowledgements

The authors would like to thank Dr. Nagabhushan KH, Vice-president, Medical Services, Microlabs Pharmaceuticals, Bangalore, Karnataka, India, for providing ATV and SMV drugs. The authors also express their gratitude to Dr. NG Nanjundaswamy, Professor and Head and Miss Shiny (PG student), Department of Pharmaceutics, Government College of Pharmacy, Bangalore, India, for helping us in preparing ATV, SMV and placebo gels. The authors acknowledge Mr. Gurinder Singh, Statistician, Chandigarh, India, for preparing the statistics.

## Authors’ contributions

The study was designed by ARP. All the clinical operations were performed by SM, while all clinical and radiographic examinations were carried out by MK and DKS, respectively. SPS and AD performed all the data evaluation and manuscript drafting. All authors read and approved the final manuscript.

## Funding

Study was self-funded and was not funded by any institution or organization.

## Competing interests

The authors declare no competing interests with regards to the authorship and/or publication of this article.

## Ethics approval

The study was approved by the Institutional Ethical Committee and Review Board, Government Dental College and Research Institute, Bangalore, India.
